# Lower serum uric acid level strongly predict short-term poor functional outcome in acute stroke with normoglycaemia: a cohort study in China

**DOI:** 10.1186/s12883-017-0793-6

**Published:** 2017-02-01

**Authors:** Shuolin Wu, Yuesong Pan, Ning Zhang, Wang Yong Jun, Chunxue Wang, On Behalf of the Investigators for the Survey on Abnormal Glucose Regulation in Patients With Acute Stroke Across China (ACROSS-China)

**Affiliations:** 1 Department of Neuropsychiatry and Behavioral Neurology and Clinical Psychology, Beijing Tiantan Hospital, Capital Medical University, Beijing, China; 2 China National Clinical Research Center for Neurological Diseases, Beijing, China; 3 Department of Neurology, Beijing Tiantan Hospital, Capital Medical University, Beijing, China; 4 Center of Stroke, Beijing Institute for Brain Disorders, Beijing, China; 5 Beijing Key Laboratory of Translational Medicine for cerebrovascular Disease, Beijing, China

**Keywords:** Uric acid, Diabetes mellitus, Stroke, Risk factors

## Abstract

**Background:**

Conflicting results on the correlation between hyperuricemia and the prognosis of stroke had been reported and the close association between serum uric acid (SUA) and abnormal glycomatabolism might further complicate the assessment of the correlation. We aimed to investigate SUA in predicting the prognosis of acute stroke in different glycometabolism status.

**Methods:**

A total of 2907 patients aged from 18 to 85 (1220 diabetes mellitus (DM), 777 prediabetes and 910 normoglycemia) were selected from the Abnormal Glucose Regulation in Patients with Acute Stroke across China (ACROSS-China) study. The patients were divided into groups according to the SUA quartile as well as decile. The correlations between SUA and the poor outcome (mRS > 2) at discharge were assessed stratified by glucose metabolism status. Multivariate logistic regression was used to analyze the potential risk factors of poor in-hospital outcome of stroke and the risk-adjustment of the correlation between SUA and the prognosis of stroke. *P <* 0.05 was considered statistically significant.

**Results:**

SUA were divided first as Quartile1 to 4 (Quartile1 < 221 μmol L^−1^; Quartile2 (221–286) μmol L^−1^; Quartile3 (286–352) μmol L^−1^ and Quartile4 > 352 μmol L^−1^), then as decile1 to 10. In normoglycaemia, SUA quartiles, deciles and continuous SUA concentration were independently significantly associated with poor outcome. Q1 was independently associated with the higher possibility of poor functional outcome (compared to Q4, odds ratios (ORs) with 95% confidential interval (CI) was 3.79 (1.23–8.67) in Q1); Lower level of SUA in DM was also associated with poor functional outcome at discharge compared to the highest level of SUA(Q4)(OR with 95% CI, 2.07 (1.05–4.08)), however, lower SUA level was also related to severer stroke at admission in DM as well as in prediabetes (*P <* 0.001 in DM and 0.023 in prediabetes) and severer stroke resulted in worse functional outcome at discharge (OR with 95% CI, 12.15 (8.08–18.21) in DM and 11.58 (7.50–23.25) in prediabetes). But in normoglycamic stroke, SUA levels did not differ in stroke severity at admission (*P =* 0.066).

**Conclusions:**

Low SUA level (<221 μmol L^−1^) independently and strongly predicts the short-term poor functional outcome in acute stroke with normoglycaemia other than diabetes or prediabetes.

**Electronic supplementary material:**

The online version of this article (doi:10.1186/s12883-017-0793-6) contains supplementary material, which is available to authorized users.

## Background

The association between hyperuricemia and cardiovascular disease has been well documented for a very long time. However, conflicting results on the correlation between hyperuricemia and the incidence and prognosis of stroke had been reported. In a meta-analysis study, Kim, et al. reported that hyperuricemia increased the risk of both the ischemic stroke and the hemorrhagic stroke as well as the risk of post-stroke mortality [1]. Several other groups also found that the post-stroke hyperuricemia could significantly exacerbate the outcome of stroke [2–4]. Other study suggested that the effect of pre-stroke hyperuricemia on poor post-stroke outcome, especially in diabetic patients, was still questionable [5]. Holme and his group also suggested that hyperuricemia might be rather a complementary indicator than an independent risk factor of AS [6]. Kramer et al. found that the importance of the pre- cardiovascular disease hyperuricemia in predicting cardiovascular disease mortality was only significant in the pre-diabetic and type 2 diabetic patients but not in the normoglycemic patients [7]. Some investigators even found that post-stroke hyperuricemia might have played a protective role in the prognosis of stroke [8–12]. Miedema et al. found no evidence to support the association between the SUA level and the outcome of stroke in their study [13].

Hyperuricemia, hyperglycaemia and cardiovascular disease are found to be co-morbid disease [14]. Hyperuricaemia is also positively associated with prediabetes diagnosed according to impaired fasting glucose criteria other than subjects with normal fasting glucose [15]. A meta-analysis also ascertain that hyperglycaemia is contributed to the development of diabetes and impaired fasting glucose [16]. Even in normal range of SUA, uric acid is related to diabetes onset in healthy lean women [17]. Since the close and complicated association between SUA and prediabetes as stated above and diabetes was a well-known risk factor of stroke [18], it is in very need to assess and compare the effect of SUA on stroke prognosis stratified by different glycometabolism status. Newman et al. reported that a combination of the post-stroke hyperuricemia and diabetes could significantly increase the risk of adverse events, including recurrent stroke, cardiovascular events even death, in post-stroke patients [3]. In another study, Zhang et al. found that the post-stroke hyperuricemia was associated with better short-term outcome of stroke in non-diabetes patients [12]. However they failed to distinguish between the pre-diabetes and the normoglycemia patients in their study. In our study, the association between SUA and the outcome of acute primary stroke would be assessed in both diabetes and normoglycemia patients. The pre-diabetes patients were excluded in our study.

## Methods

### Patient selection

ACROSS-CHINA” was a large prospective cohort study conducted in China from 2008 to 2009 that investigated on abnormal glucose regulation in acute first-ever stroke (within 14 days), in which a total of 3450 patients with ischemic stroke, intracerebral hemorrhage (ICH) or subarachnoid hemorrhage (SAH) were successively recruited from 34 hospitals across China [19, 20]. Acute stroke was diagnosed according to World Health Organization combined with CT or MRI confirmation [21].

**Fig. 1:**
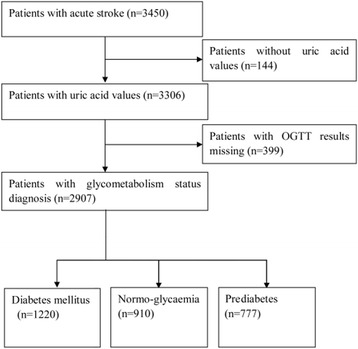
Flow chart of patient selection. OGTT, oral glucose tolerance test

All the patients without previous DM were required to perform a standard oral glucose tolerance test (OGTT) at the day 14 ± 3 after stroke onset or before discharge according to the World Health Organization criteria [22]. Patients were categorized as diabetes, pre-diabetes or normoglycemia according to their OGTT results. One hundred and forty-four patients without SUA values and 399 patients with missing OGTT results were excluded respectively. In the remaining 2,907 patients, there were 1220 diabetics, 777 prediabetics and 910 normoglycaemic patients (Fig. 1).

### Data collection

Demographic, education degree, tobacco use and alcohol consumption, physical exam and a National Institute of Health Stroke Scale (NIHSS) score were obtained at admission. A detailed history of previous diseases, such as hypertension, coronary artery disease and atrial fibrillation, were also recorded. Body mass index (BMI) was defined as body weight (kg)/the square of height (m). Participants without a history of diabetes were required to take an OGTT test within 24 h after the hospital admission. The lab tests included blood routine test, high density lipoprotein, low density lipoprotein, creatinine, SUA, homocysteine, glucose and insulin level, et al. All the lab tests were performed within 24 h after admission at fasting condition by the certified central laboratory. SUA was measured by the urine enzyme endpoint method. The index of insulin resistance was calculated with the Homeostasis Model Assessment 2- Insulin Resistance (HOMA2-IR) method [23]. A Modified Rankin Scale (mRS) was calculated in every participant at discharge to evaluate the in-hospital outcome of stroke.

### The criteria of diagnosing abnormal glycometabolism

The OGTT result and the blood glucose level were used to divide patients into three categorise based on the 1999 World Health Organization (WHO) criteria. The details were as follow: (1) Diabetes mellitus (DM) was defined as fasting plasma glucose (FPG) ≥ 7.0 m mol L^−1^ and/or 2-h value (2-h-value) ≥ 11.1 m mol L^−1^; (2) Pre-diabetes consisted of both impaired glucose tolerance (IGT) and impaired fasting glucose (IFG). IGT was defined as FPG < 7.0 m mol L^−1^ and a 2-h-value between 7.8 mmol/L and 11.1 m mol L^−1^. IFG was defined as fasting glucose value between 6.1 m mol L^−1^ and 7.0 m mol L^−1^ with a normal 2-h-value; (3) normoglycemia was defined as FPG < 6.1 m mol L^−1^ and a 2-h-value < 7.8 m mol L^−1^. Patients who had a history of DM or had ever received hyperglycemic agents for at least 6 months were also assigned to the DM group. All the prediabetics were newly-diagnosed.

### Other variables definition

Tobacco use was categorized as ‘current’, ‘previous’ or ‘never’ smoking. ‘Current smoking’ was defined as that the individual was an active smoker at the time of the stroke. ‘Previous smoking’ was defined as that the individual had quit smoking 1 year prior to the stroke. ‘Never smoking’ was defined as that the individual had never smoked prior to the stroke. Alcohol consumption was categorized as ‘never drinking’, ‘mild drinking’,‘moderate to severe drinking’. ‘Moderate or severe drinking’ was defined as that the individual consistently consumed ≥2 standard size alcoholic beverages per day. ‘Mild drinking’ was defined as that the individual consumed <2 standard size alcoholic beverages per day. ‘Never drinking’ was defined as that the individual had never consumed any alcoholic beverages. Stroke severity was categorized by the NIHSS score as mild stroke (NIHSS < 9) or severe stroke (NIHSS ≥ 9) [4].

### Outcome assessment

The primary endpoint comprised death and dependency with mRS >2 [24, 25]. Death (mRS = 6) was recorded within 14 ± 3 days after admission to the hospital. Dependency (mRS = 3 to 5) was assessed at 14 ± 3 days after the index date of hospital admission or at hospital discharge. Death indicated all-cause death according to the International Classification of Diseases, 10th Revision. Neurological improvement and deterioration were used as supplemental indicators to describe the association between SUA and stroke outcome. Neurological improvement was defined as 4 point decrease in NIHSS during hospitalization or a 0 point status on NIHSS at discharge. Neurological deterioration was defined as 1 point increase in NIHSS during hospitalization [24, 26, 27].

### Statistical analysis

Baseline clinical features were compared stratified by different diabetes diagnosis and functional outcome. All continuous variables were presented as mean ± standard deviation for normal distribution and median (Quartile 1 to 3) for skewed distribution. Kolmogorov-Smirnov test was used for normal distribution test. Categorical variables were presented as frequencies and/or prevalence/incidence rate. For the continuous variables, when the group numbers were more than two, if the variable was normal distribution, one-way ANOVA test was used to perform the comparison (LSD-test for variables with homogeneity of variance and Games-Howell test for variables with heterogeneity of variance), otherwise Kruskal-Wallis H test was performed instead; when the group numbers were just two, if the variable was normal distribution, *T*-test was used to perform the comparison, otherwise Mann–Whitney *U* test was used instead. For categorical variables, the comparisons were used Chi-square test and Fisher exact test. The adjustment of the correlation between different variables was done by multivariate logistic regression. Variables with a *P* value < 0.1 in univariate analysis were included in the multivariate regression test. Variables, which had shown potential association to the outcome of stroke during the literature search, were also included in the multivariate regression test. The independent factors included in the multivariate analysis were age, gender, education degree, tobacco use, alcohol consumption, BMI, high density lipoprotein, low density lipoprotein, creatinine, systolic and diastolic blood pressure, HOMA2-IR, homocysteine, a history of coronary heart disease, a history of hypertension, a history of atrial fibrillation, stroke types and stroke severity at admission. The use of Aspirin, Diuretics and Coumadin [28], which could influence SUA concentration, were also analyzed. SUA was compared between “use” and “non-use” of them respectively and stratified by different glycometabolism first, then it will be recognized as a confounding factor if *P <* 0.05 and entered into the according multivariate analysis. The fitness of the models was evaluated by using the Hosmer and Lemeshow goodness-of-fit test and a P value > 0.2 was considered as a good fit. All the statistical analyses were done with SPSS ver. 19.0 software (SPSS Inc., Chicago, IL). A *P <*0.05 was considered statistically significant.

## Results

### Comparison of the characteristics of patients in different glucose **metabolism groups**

Patients with normoglycemia accounted for 31.3% of the study population. The differences in the characteristics of patients among different groups were shown in Table 1. The normoglycemia group had highest percentage of males (67.7% vs. 64.5%vs. 58.3%, *P <* 0.001) and higher prevalence of current smoking (*P =* 0.036). The mean level of age in this group was significantly younger than that of the DM or prediabetes group (*P <* 0.001). In the physical exam and the lab test, the patients in the normoglycemia group had significantly lower blood pressure, BMI, NIHSS score, low density lipoprotein level and HOMA2-IR than that in the DM or prediabetes group (all *P <* 0.05). The high density lipoprotein, homocysteine in the normoglycemia group were significantly higher than that in the DM or prediabetes group (all *P <* 0.05). Previous histories of coronary heart disease, hypertension or atrial fibrillation in thenormoglycaemia group were significantly less common than that in the DM group (all *P <*0.05). Ischemic stroke was the most common type of stroke in all groups, followed by intracerebral hemorrhage and SAH. The percentages of stroke types were significantly different in patients with different glycometabolism status (*P <*0.001).

**Table 1:** Baseline Clinic features according to glycometabolism diagnosis by OGTT

Clinic features	Diabetes mellitus (*n =* 1220)	Prediabetes (*n =* 777)	Normoglycemia (*n =* 910)	*P*
Age, y (median, Q_1–3_)	64 (55, 73)	61 (52,72)	58 (49, 69))	<0.001
Male, *n* (%)	711(58.3)	501 (64.5%)	616 (67.7)	<0.001
Tobacco use, *n* (%)				0.036
Never	712 (58.4)	428 (55.1)	480 (52.7)	
Quit	110 (9.0)	74 (9.5)	79 (8.7)	
Current	351 (28.8)	246 (31.7)	320 (35.2)	
Alcohol consumption, *n* (%)				0.164
Mild	213 (17.5)	132 (17.0)	171 (18.8)	
Moderate to severe	165 (13.5)	131 (16.9)	152 (16.7)	
Never	817 (67.0)	494 (63.6)	564 (62.0)	
BMI, kg/m^2^ (median, Q_1–3_)	25.0 (22.9,27.3)	24.5 (22.5,26.8)	24.2 (22.0,26.2)	<0.001
NIHSS (median,Q_1_-Q_3_)	5 (2–9)	5 (2,9)	4 (2–8)	0.002
Creatinine,m mol L^−1^ (median, Q_1–3_)	71.2 (58.0, 87.0)	72.0 (60.0, 87.0)	71.8 (59.9, 86.7)	0.425
LDL, m mol L^−1^ (median, Q_1–3_)	3.0 (2.4, 3.6)	2.9 (2.3, 3.5)	2.8 (2.3, 3.4)	0.001
HDL,m mol L^−1^(median, Q_1–3_)	1.1(1.0, 1.3)	1.2 (1.0, 1.4)	1.2 (1.1, 1.4)	<0.001
Systolic blood pressure, mmHg (median, Q_1–3_)	150 (138, 160)	143 (130, 160)	146 (130, 160)	<0.001
Homocysteine, μ mol L^−1^, (median, Q_1–3_)	14.4 (11.5, 18.7)	15.5 (12.0, 21.9)	15.7 (12.3, 22.8)	<0.001
HOMA2-IR (median, Q_1–3_)	2.5 (1.6, 4.0)	1.9 (1.1, 3.0)	1.6 (0.9, 2.5)	<0.001
Past medical history, yes, n (%)				
Coronary artery disease	172 (14.1)	87 (11.2)	90 (9.9)	0.009
Hypertension	795 (65.2)	470 (60.5)	514 (56.5)	<0.001
Atrial fibrillation	66 (5.4)	54 (6.9)	31 (3.4)	0.006
Stroke types, *n* (%)				<0.001
IS	1015 (83.2)	541 (69.6)	675 (74.2)	
ICH	174 (14.3)	185 (23.8)	198 (21.8)	
SAH	31 (2.5)	51 (6.6)	37 (4.1)	

*OGTT* the oral glucose tolerance test, *BMI* body mass index, *LDL* low density lipoprotein, *HDL* high density lipoprotein, *NIHSS* the National Institutes of Health Stroke Scale, *HOMA2-IR* the correctly solved computer model for homeostasis model assessment of insulin resistance, *IS* ischemic stroke, *ICH* intra-cerebral hemorrhage, *SAH* subarachnoid hemorrhage

### Comparison of the characteristics of patients with different functional outcomes of the stroke

There were no significant differences between the favorable outcome group and the poor outcome group in the mean level of age, BMI, HOMA2-IR, low density lipoprotein level, high density lipoprotein level, the prevalence of the histories of coronary heart disease or hypertension. Compared to the favorable outcome group, the mean level of creatinine was significantly lower in the poor outcome group, whereas the blood pressure and homocysteine level were significantly higher in the poor outcome group. The percentage of moderate to severe alcohol consumption as well as the percentage of severe stroke and atrial fibrillation in the poor outcome group was significantly higher than that in favorable outcome group. The percentage of mild drinking was significantly higher in favorable outcome group than that in the poor outcome group. Ischemic stroke was the most common type of stroke in both groups. Cardioembolic ischemic stroke was only accounted for 5.7% (*n =* 91) of the total patients with poor functional outcome. The intracerebral hemorrhage was more commonly seen in the poor outcome group than that in the favorable outcome group while ischemic stroke and SAH were more prevalent in the favorable outcome group. DM was more prevalent in the poor outcome while normoglycemia were more commonly seen in the favorable outcome group (Table 2).

**Table 2:** Baseline Clinic features according to poor functional outcomes evaluated by mRS > 2

Clinic features	Favorable outcomes (*n =* 1305)	Poor functional outcomes (*n =* 1602)	*P*
Age, y	60 (52, 71)	62 (53, 72)	0.003
Male, *n* (%)	954 (73.1)	1089 (68.0)	<0.001
Tobacco use, *n* (%)			0.036
Never	692 (53.0)	928 (57.9)	
Quit	126 (9.7)	137 (8.6)	
Current	436 (33.4)	481 (30.0)	
Alcohol consumption, *n* (%)			0.006
Never	811 (62.1)	1064 (66.4)	
Mild	266 (20.4)	250 (15.6)	
Moderate to severe	192 (14.7)	256 (16.0)	
BMI(kg/m^2^)	24.6 (22.5,26.7)	24.5 (22.4,26.8)	0.637
NIHSS scales			<0.001
<9	1256 (96.2)	996 (62.2)	
9–18	30 (2.3)	474 (29.6)	
> 18	7 (0.5)	124 (7.7)	
Creatinine (m mol L^−1^)	72.7 (60.0, 87.5)	70.0 (57.5,85.0)	0.002
LDL(m mol L^−1^)	2.8 (2.3,3.5)	2.9 (2.3,3.5)	0.290
HDL(m mol L^−1^)	1.2 (1.0,1.4)	1.2 (1.0,1.4)	0.125
Systolic blood pressure (mmHg)	140 (130,160)	150 (134,161)	<0.001
Homocysteine (μmol L^−1^)	14.6 (11.4,19.8)	15.8 (12.3,21.4)	<0.001
HOMA2-IR	1.8 (1.1,3.0)	2.0 (1.2,3.1)	0.055
Past medical history, yes, *n* (%)			
Coronary heart disease	146 (11.2)	203 (12.7)	0.230
Hypertension	785 (60.2)	994 (62.0)	0.296
Atrial fibrillation	53 (4.1)	98 (6.1)	0.013
Stroke types, n (%)			<0.001
Ischemic stroke	1059 (81.1)	1172 (73.2)	<0.001
Cardioembolic	44 (3.4)	91 (5.7)	
Large-artery atherosclerosis	565 (43.3)	819 (51.1)	
Small-artery occlusion Lacunar	367 (28.1)	200 (12.5)	
Other demonstrated or undemonstrated	38 (2.9)	25 (1.6)	
ICH	176 (13.5)	381(23.8)	
SAH	70 (5.4)	49 (3.1)	
Glycometabolism status			**<0.001**
Diabetes mellitus	500 (38.3)	720 (44.9)	
Prediabetes	348 (26.7)	429 (26.8)	
Normoglycaemia	457 (35.0)	453 (28.3)	

*mRS* modified Rankin scale, *BMI* body mass index, *LDL* low density lipoprotein, *HDL* high density lipoprotein, *NIHSS* the National Institutes of Health Stroke Scale, *HOMA2-IR* the correctly solved computer model for homeostasis model assessment of insulin resistance, *IS* ischemic stroke, *ICH* intra-cerebral hemorrhage, *SAH* subarachnoid hemorrhage

### Analysis on medication use and SUA concentration

There are 1542 patients with data on aspirin, 20 patients on Coumadin and 124 patients on Diuretics. SUA levels were compared between “use” and “non-use” of them respectively and stratified by different glycometabolism. After that a significant association was found between Aspirin use and SUA level among normal glycemic stroke patients (Additional file 1: Table S3, Additional file 2: Table S4 and Additional file 3: Table S5). Aspirin use was then entered into the multivariate analysis on SUA and the outcome among normoglycaemic stroke patients.

### Correlation between SUA and poor functional outcome at discharge

#### Univariate analysis of SUA quartiles on poor outcome

**Fig. 2:**
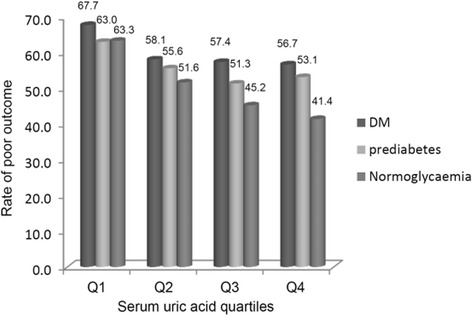
Poor outcome rates depending on serum uric acid quartiles stratified by glycometabolism status. OGTT, the oral glucose tolerance test. Q1-4 indicates serum uric acid levels by quartiles. Q1 to Q4 were Q1, <221 μmol L^−1^; Q2, (221–286) μmol L^−1^; Q3, (286–352) μmol L^−1^ and Q4, >352 μmol L^−1^

The patients were divided into quartile groups (Q1 to Q4) based on their SUA level. The concentrations of SUA in these groups were as follow: Q1 < 221 μmol L^−1^, Q2 221-286 μmol L^−1^, Q3 286-352 μmol L^−1^ and Q4 > 352 μmol L^−1^ (DM group, N_Q1=_307, N_Q2_ = 306, N_Q3_ = 310, N_Q4_ = 291; Prediabetes group, N_Q1=_184, N_Q2_ = 198, N_Q3_ = 190, N_Q4_ = 198; Normoglycaemia group, N_Q1=_229, N_Q2_ = 218, N_Q3_ = 211, N_Q4_ = 248). In both diabetic and normoglycemic strokes, the occurrence of stroke poor outcome decreased across the increasing SUA levels from Q1 to Q4(*P =* 0.019 in DM, 0.016 in normoglycemia), however, it didn’t differ in SUA quartiles in prediabeteic strokes (*P =* 0.110 in prediabetes) (Fig. 2).

### Multivariate analysis of SUA quartiles on poor functional outcome at discharge

To further evaluate the correlation between the SUA level and the poor post-stroke outcome, the SUA level along with other potential risk factors of poor post-stroke outcome were introduced into a regression model as independent variables and the multivariate logistic regression was used to assess the adjusted correlation between the SUA level and the outcome of stoke. The NIHSS score was entered into the regression model as an ordinal variable with the cut-off point of 9 first and then as a continuous one.

**Fig. 3:**
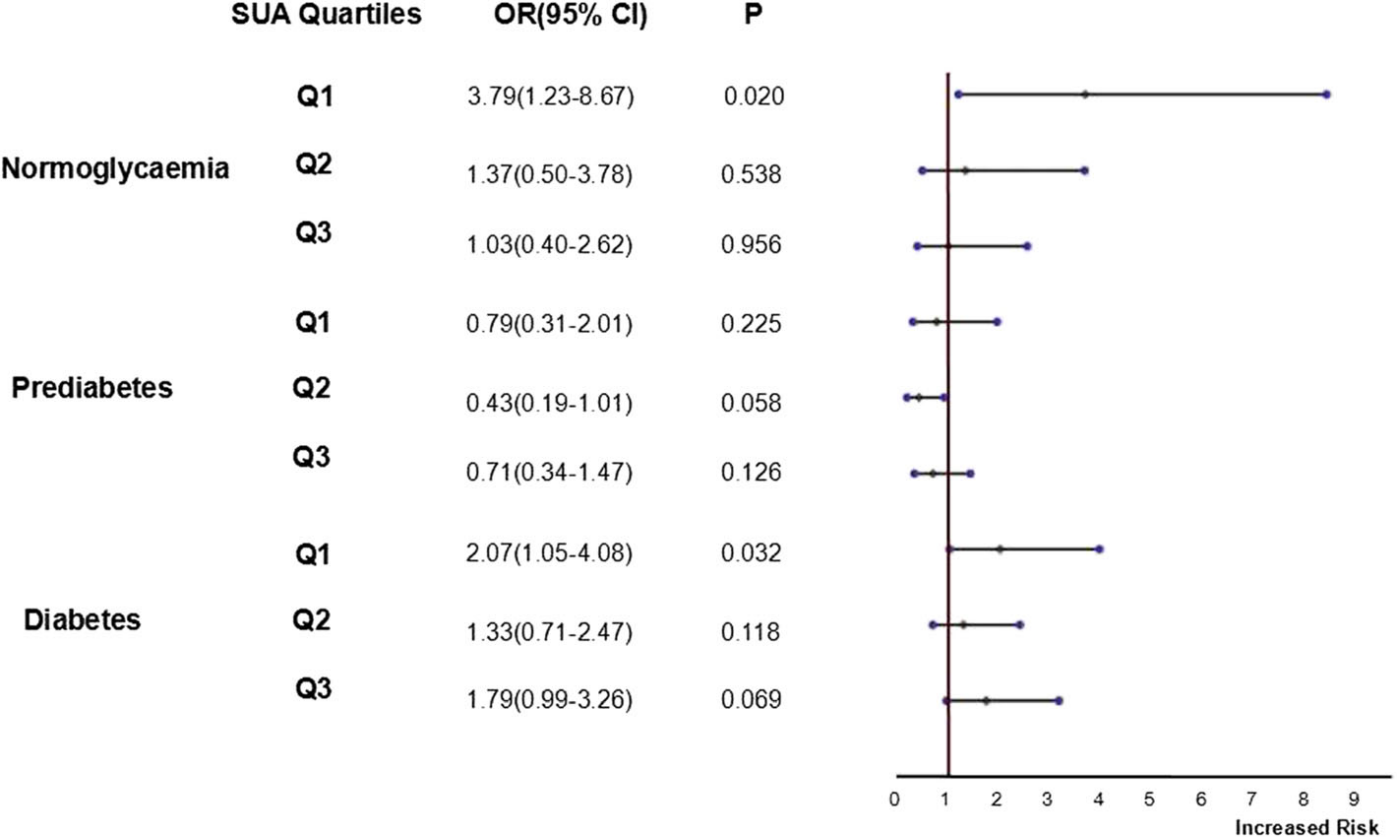
Association between uric acid quartiles and poor outcomes in multivariate logistic regression analysis ^a^ (^**a**^ adjusted for age, gender, stroke types, tobacco use, alcohol consumption, education level received, a history of hypertension, coronary artery disease or atrial fibrillation, NIHSS <9 VS ≥9, body mass index, homocysteine, systolic blood pressure, diastolic blood pressure, HOMA-IR, low density lipoprotein, high density lipoprotein, creatinine. In normoglycaemic subjects, Aspirin use was included as a binary variable in the analysis). NIHSS indicates the National Institutes of Health Stroke Scale; HOMA2-IR indicates the correctly solved computer model for homeostasis model assessment of insulin resistance

The final results of the regression showed that there was a negative correlation between the SUA level and the occurrence rate of poor outcome of stroke in normoglycemic group. In the normoglycemic group, the occurrence rate of poor outcome in Q1 group was also significantly higher than that in the Q4 group (OR = 3.79, 95% CI 1.23–8.67, *P =*0.020) (Fig. 3); In DM group, the possibility of poor outcome in lowest SUA group (Q1) was significantly higher than that in Q4 (OR = 2.07, 95% CI 1.05–4.08). The possibility of poor outcome in Q2 and Q3 groups were not significantly higher than that in Q4 (Q2: OR = 1.33, 95% CI 0.71–2.47; Q3: OR = 1.79, 95% CI 0.99–3.26). In prediabetes, the possibility of poor outcome did not differ in SUA quartiles (all *P >* 0.05). The Hosmer and Lemeshow goodness-of-fit test results (*χ* ^2^, P) were (8.839, 0.356), (110.982, 0.215) and (4.558, 0.755) in the DM, prediabetes and the normoglycemia group, respectively (Fig. 3).

In normoglycaemic stroke patients, NIHSS was then entered as a continuous variable into multivariate analysis instead of NIHSS as a binary variable. The association was still significant after the new adjustment (Additional file 4: Table S7).

In normoglycaemic stroke patients, further analysis was performed stratified by sex (Additional file 5: Table S6). There was 620 male and 290 female in normoglycaemic group. In male, after adjusted for the same variables as the above, the lowest level of SUA is still an independent risk factor for the poor functional outcome (OR with 95% CI 5.06(1.17–12.05), *P =* 0.031). However it was not the case in female patients (OR with 95% CI 9.21(0.98–38.66), *P =* 0.158).

### Multivariate analysis of SUA deciles on poor functional outcome

**Fig. 4:**
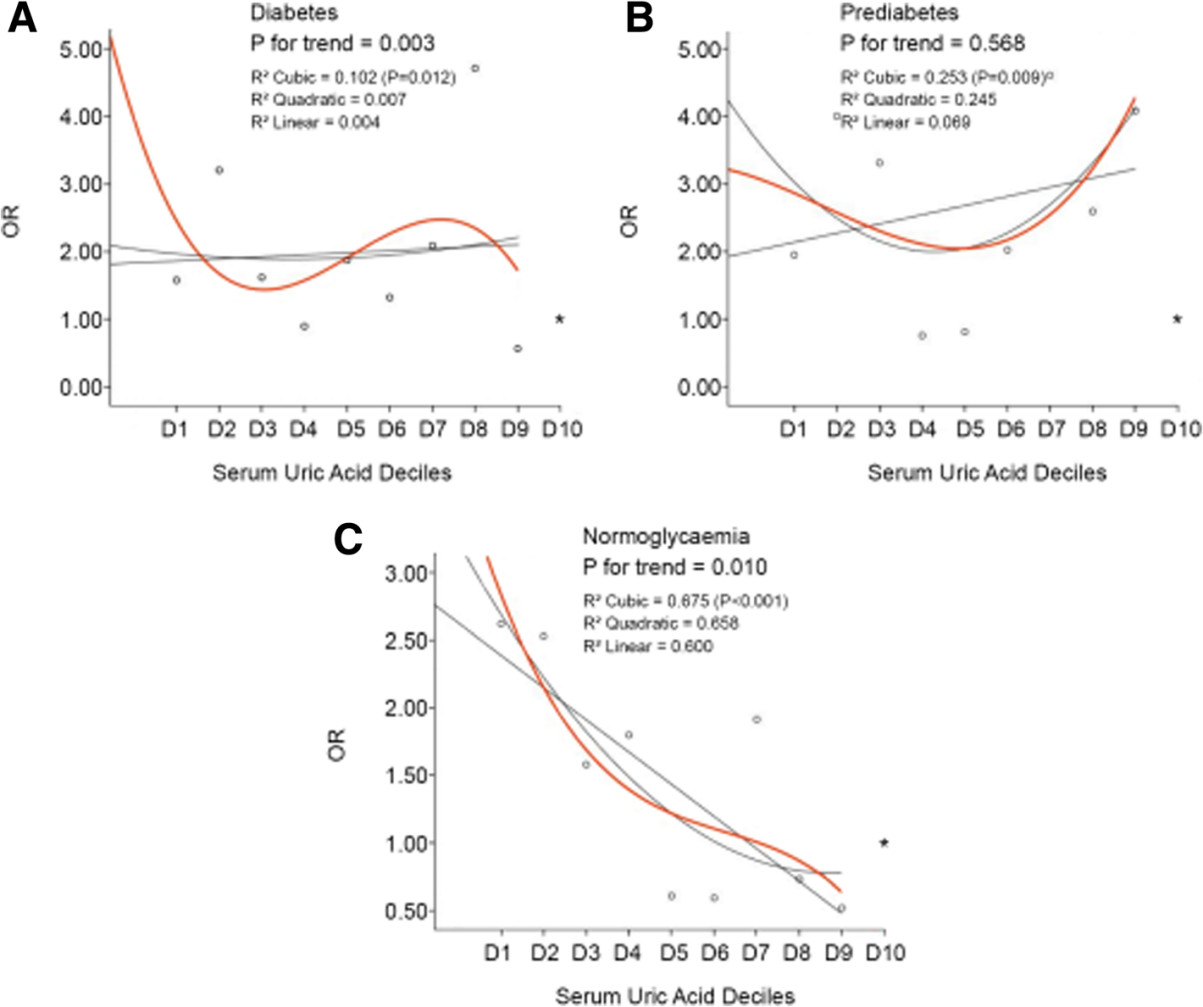
Association between serum uric acid deciles and poor outcomes in multivariate logistic regression analysis^a^ (^**a**^ adjusted for the same variables as those in Fig. 3). *indicates the reference OR (the OR of D10 = 1). Reference point not included as basis for fit of regression lines. D1-10 indicates 1st decile to the 10th decile of serum uric acid. D1 to D10 (μmol L^−1^) in ‘Diabetes’ were <169, 169–206, 206–235, 235–261, 261–286, 286–308, 308–334, 334–368, 368–424, >424. D1 to D10 (μmol L^−1^) in “Prediabetes” were <155, 155–208, 208–237, 237–266, 266–287, 287–314, 314–342, 342–380, 380–438, >438. D1-10 (μmol L^−1^) in ‘Normoglycaemia’ were <151, 151–201, 201–235, 235–260, 260–288, 288–316, 316–343, 343–381, 381–425, >425

SUA was re-divided into decile groups (D1 to D10) in different glucose metabolism groups. The SUA quartile groups in previous regression model were replaced with SUA deciles and a new regression model was calculated. The ORs of the incidence rate of poor outcome obtained from the regression model were used in the curve-fitting analysis. The curve-fitting results showed a U-shape curve and the cubic curve had the highest R^2^ value in both the DM group and the normoglycemia group (Fig. 4a and c). The occurrence of poor outcome significantly decreased across SUA deciles from D1 to D9 in both normoglycemia and DM strokes (P for trend0.003 and 0.010, respectively), while it did not differ from D1-10 in prediabetes group (Fig. 4 b, P for trend, 0.568).

### Multivariate analysis of continuous SUA concentration on poor functional outcome

SUA were further reintroduced into the multivariate analysis as a continuous variable. After adjusting for the same confounding factors as the above, higher SUA was still significantly independently associated with the less possibility of poor outcome in patients with normoglycaemia, while the association did not exist in patients with DM or prediabetes (Table 3). The Hosmer and Lemeshow goodness-of-fit test results (*χ* ^2^, P) were (9.873, 0.274) in DM, (4.719, 0.787) in prediabetes and (5.621, 0.690) in normoglycemia group, respectively.

**Table 3:** Association between SUA as a continuous variable and poor outcome stratified by glycometabolism status^a^

Glycometabolism status	Odds ratio with 95% confidence intervals	*P*
Diabetes mellitus	0.98 (0.97–1.00)	0.135
Prediabetes	1.00 (1.00–1.01)	0.105
Normoglycaemia	0.99 (0.98–1.00)	0.022

^a^adjusted for the same variables as those in Fig. 3

### SUA concentration and Neurological functional change stratified by glycometabolism

#### SUA and Neurological improvement

In diabetic and prediabetic stroke patients, SUA quartiles were significantly associated with neurological improvement. Patients with the lowest level of SUA (<221umol/L) occupied 28.5% and 30.6% within the diabetes and prediabetes being neurological improved (both *P <*0.05. Additional file 6: Table S1. Additional file 7: Figure S1).

#### SUA and Neurological deterioration

There were no significant association between SUA and neurological deterioration in all the glycometabolism statuses (Additional file 8: Table S2. Additional file 9: Figure S2).

### Correlation between SUA levels and stroke severity at admission

**Fig. 5:**
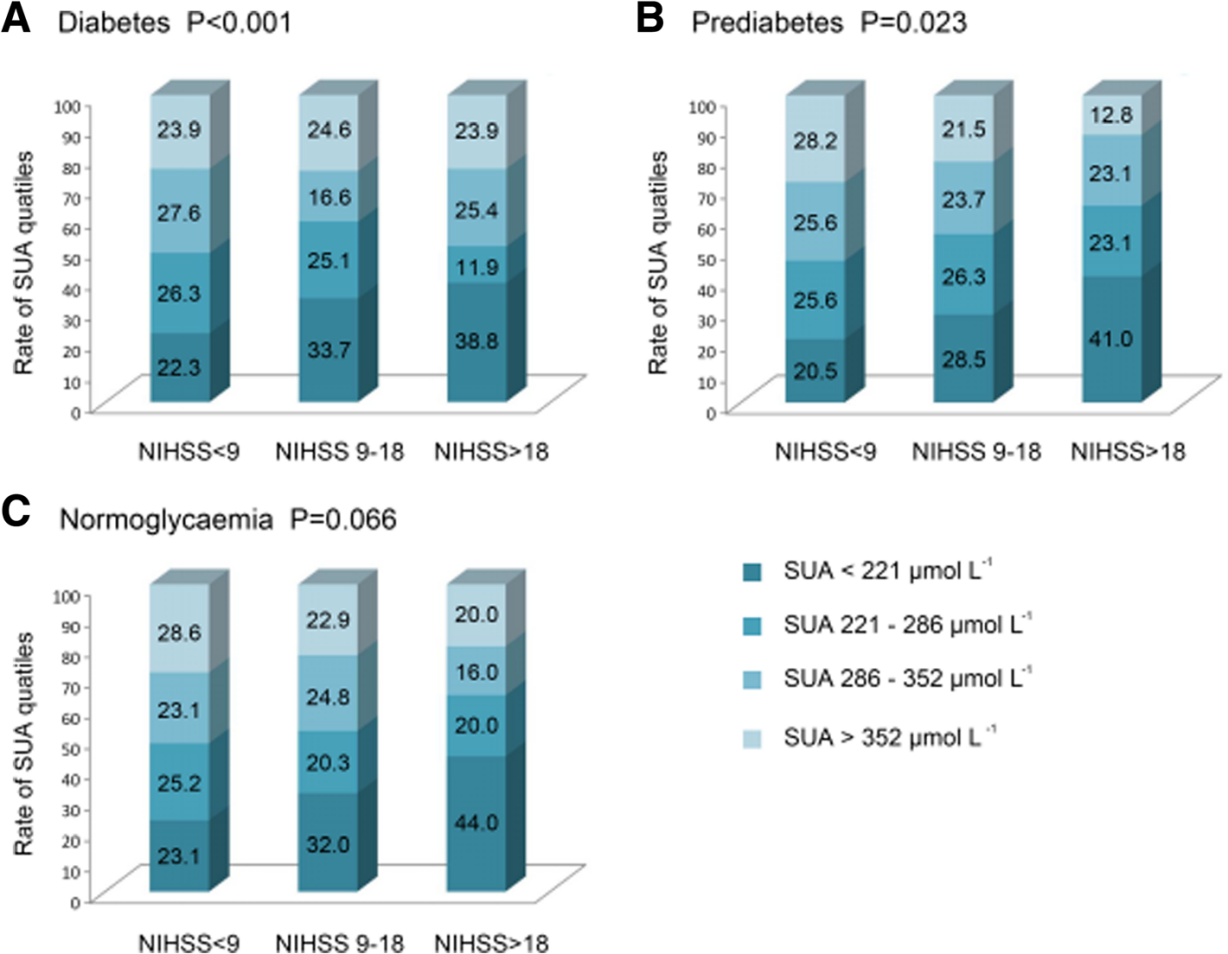
Association between serum uric acid levels and stroke severity stratified by glycometabolism status. SUA indicates serum uric acid. NIHSS indicates the National Institutes of Health Stroke Scale

The correlation between stroke severity (NIHSS < 9, 9–18,>18) and the SUA level was also analyzed. The patients were divided into 3 groups according to NIHSS of <9, 9–18 and >18, respectively. The correlation between stroke severity and the SUA quartile was calculated (Fig. 5). In DM and prediabetes, the percentage of patients with low SUA (Q1) in the severe stroke group (NIHSS > 18) was significantly higher than that in the mild stroke group (NIHSS < 9) (*P <* 0.001for DM and 0.023 for prediabetes). There were no significant differences in the SUA level across different stroke severity in the normoglycemia group (*P =* 0.066).

### Other variables associated with the poor functional outcome of the stroke in multivariate analysis

NIHSS ≥9 was a significant risk factor of the poor outcome in all patients (In diabetes, 12.15 (8.08–18.21); In prediabetes, 11.58 (7.50–23.25); In normoglycemia, 18.40 (7.90–25.45)). In normoglycemia group, higher BMI as well as higher systolic blood pressure were also related to significantly higher risk of poor outcome (BMI 1.09 (1.02–1.18), systolic blood pressure 1.02 (1.01–1.18)).

## Discussion

Most of previous studies suggested that hyperuricemia was a risk factor of stroke. In the present study, SUA values in predicting poor functional outcomes after stroke differed in 3 glycometabolism statuses. Lower SUA level (SUA < 220 μmol L^−1^) was an independent risk predictor of the short-term prognosis of the acute first-ever stroke in patients with normoglycemia other than DM or prediabetes and such an effect might has gender difference. In the present study, the relationship is much more robust in male than in female normoglycaemic stroke patients.

### Difference in the association of SUA level and post-stroke poor functional outcome among different glycometabolism status

The correlation between the SUA level and the poor post-stroke outcome in the normoglycemia patients was different from that in the DM or prediabetes patients in our study. Low SUA level was an independent predictor of the poor outcome of stroke in the normoglycemia patients but not in DM or prediabetes group. In DM group, a paradox association was found: 1) the association between the lowest SUA quartile (Q1) and the poor outcome was observed as significant (Fig. 3. *P =* 0.032) but the trend from Q1-Q4 was not (Fig. 3. P for trend = 0.109); 2) the association between SUA deciles and poor outcome was in a significant trend (Fig. 4 a. P for trend 0.003); 3) however, the significant association disappeared when assessment was performed between the continuous SUA concentration and the poor outcome (Table 3 . *P =* 0.135). In prediabetes group, possibility of poor outcome in the lower SUA levels was similar to that in the higher SUA levels either SUA was introduced into the multivariate analysis as a categorical variable or a continuous variable.

These debated results might be attributed to the negative association between SUA levels and stroke severity in DM and prediabetes (Fig. 5 a and b. *P <* 0.001 for DM and 0.023 for prediabetes). The percentage of the lower SUA level was significantly increased across the aggravated stroke severity in diabetes and prediabetes group, and stroke severity (measured as NIHSS level) was also significantly associated with the poor outcome in the multivariate analysis, we speculated that the effect of the low SUA level in DM on the poor outcome of stroke might be carried out via its correlation with the severity of stroke. On the contrary, based on the fact that there was no correlation between the SUA level and the severity of stroke in normoglycemia patients (Fig. 5c. *P =* 0.066), the association of SUA and poor outcome was independent of stroke severity at baseline in normoglycemia patients. Therefore, SUA in normoglycaemia was an independent risk factor of poor outcome, whereas SUA in DM was only a complementary but not an independent one.

Zhang et al. reported their findings of a similar study in 2012 [12]. The correlation between the SUA level and the poor outcome of stroke in patients with normoglycemia and DM in their study was in line with us; Although the patients in their study were young and mid-age adults (18–45 years old) which was different with us (from 18 to 85 years old), the results were similar. It is a pity that they did not differentiate the prediabetes patients from the ‘real’ normoglycemia patients. Further than their research, we examine the association in prediabetes. Though we did not found a significant association between SUA and poor outcome in prediabetes, concerning the results that the association was much more significant and prominent in normoglycaemia patients than those with abnormal glycometabolism, it was highly implied that glucose metabolism might deeply affect the influence of SUA on post-stroke short-term outcome.

Till now, the effect of SUA on the prognosis of stroke in abnormal glucose metabolism is still debatable. Kramer et al. in their 2010 study reported that hyperuricemia was a significant risk factor for the mortality of cardiovascular disease in patients with pre-diabetes and typeIIdiabetes, but it did not increase the risk of mortality in patients with normoglycemia [7]. Their findings contradicted to our results. The conflicting results might be caused by the different race (Caucasian vs. Chinese) and age (mid-age to elders vs. 18 to 85) of the participants as well as the different sampling time of SUA (pre-event vs. post-event different phases).

Gender difference is attributed to SUA concentration [29, 30], thus effect of SUA on poor functional outcome differs in gender. There is no significant association found in female subgroup in the present study, which is mainly caused by the not large enough sample size (only 290 female stroke patients with normoglycaemia).

### The possible mechanisms of the association between post-stroke SUA levels and the outcomes after stroke

There were conflicting results about the association between the post-stroke SUA level and the prognosis of stroke in the literature. Several studies reported that hyperuricemia was a risk factor for the poor outcomes [2–4] and suggested that the follow adverse effects of uric acid on the cardiovascular system might be the underline reasons: first, Hyperuricemia could cause insulin resistance [31]; second, hyperuricemia could increase the risk of hypertension and further aggravated atherosclerosis [32]; third, hyperuricemia could directly or indirectly impair the self-regulation of the arteriole and increased the risk of stroke [33].

At the meanwhile, results from other studies suggested hyperuricaemia might be a protective factor of stroke [8–12, 34]. Most of them [8, 10–12, 34] were published after the meta-analysis study from Kim, et al. [1]. In another study, administration of uric acid during the thrombolysis procedure in acute ischemic stroke patients had been proven to be neuro-protective [35]. It had also been proven in another study that low SUA was associated with larger infarction area and worse neurological deficit in the stroke patients [36]. The possible mechanisms of this protective effect of SUA included: (1) SUA was one of the most important serum antioxidant factors and an anti-inflammatory factor [37–40]; (2) SUA could stabilize endothelia progenitor cells (EPCs) [41]; (3) SUA could decrease the degradation of the extracellular superoxide dismutase-3(SOD_3_) [42]; (4) In the chronic disease, hyperuricaemia was a reaction of the negative feedback mechanism to counterbalance the increased level of reactive oxygen species (ROS) [43]. It had been proven that Xanthine Oxidase (XO) expression increased after acute ischemic cerebral lesion and the increased XO would further lead to the uric acid level in local brain tissue to increase significantly. The rate of the change in XO and local uric acid level was positively correlated with the severity of the ischemic damage of the brain tissue [44, 45]. In 2010, Brouns, et al. reported the rate of SUA decrease in the first 7 days after stroke was positively correlated with the severity of the stroke as well as the incidence rate of poor post-stroke outcome [46]. Therefore, the concentration of peripheral SUA post stroke is most possibly in a dynamically decreasing pattern but it is still unknown exactly.

This dynamically decreasing pattern of SUA post stroke would attribute to the contradictory results on SUA and poor outcome. Of all the researches, SUA values from the different measurement timing post stroke (some were within 3 days, some were within 14 days, others were within several hours, etc.) could not stand for the SUA level in a concordant post-stroke phase. Since its dynamic changes, the association of SUA and poor outcome was altered concerning a specific population (poor outcome or favorable outcome). For example, as for the patients with poor outcome, when the timing of SUA evaluated was just at the phase of higher SUA level, we might find an adverse effect of SUA on poor outcome, otherwise the effect might find to be protective. Therefore, the diverse investigations on SUA at different phases post stroke might come to paradox results. Further investigation needs to be carried out on figuring out the pattern of SUA concentration post stroke and its association as well the abnormal glucose metabolism on poor prognosis.

### Advantages and limitations

ACROSS-CHINA is a nationwide, prospective, multiple-center study. In our study, pre-diabetic patients were excluded by OGTT test result to eliminate their possible interference to the results. There were some limitations in our study. First, only patients with SUA data were included in the study and this might cause selection bias. Second, there were no data of the dynamic change of SUA level. Third, only a few data on medications that might affect the SUA level were collected and no data on non-VKA oral anticoagulants (NOACs). Fourth, lack of data on exercise pre- and post- stroke. Exercise is also a major factor to influence SUA concentration [47].

## Conclusions

The associations between post-stroke SUA and poor functional outcome at discharge differ in diverse abnormal glycometabolism populations. In acute stroke patients with normoglycemia, low SUA level (<220 μmol L-1) is an independent risk predictor of poor short-term outcome other than DM or prediabetes. The debated conclusion on the association between SUA and the poor outcome after stroke might be due to the diverse post-stroke time of blood sample for SUA in those different studies. Further research will be conducted on the association between the pattern of SUA change in stroke patients with different time of stroke onset, different glycoemtabolism status, different gender, through recording the dynamic value of SUA post stroke and the poor functional outcome and stratifying the study group by diagnosing glycometabolism status.

## Additional files

Additional file 1: Table S3. Aspirin use and SUA levels stratified by glycometabolism. (DOC 32 kb)
Additional file 2: Table S4. Coumadin use and SUA levels stratified by glycometabolism. (DOC 31 kb)
Additional file 3: Table S5. Diuretics use and SUA levels stratified by glycometabolism. (DOC 31 kb)
Additional file 4: Table S7. SUA quartile and poor functional outcome at discharge with NIHSS adjusted as a continuous variable. (DOC 28 kb)
Additional file 5: Table S6. Multivariate analysis on SUA and poor functional outcome in normoglycaemic stroke stratified by gender. (DOC 22 kb)
Additional file 6: Table S1. Uric acid level and neurological improvement stratified by glycometabolism status. (DOC 31 kb)
Additional file 7: Figure S1. (TIF 740 kb)
Additional file 8: Table S2. Uric acid level and neurological deterioration stratified by glycometabolism status. (DOC 31 kb)
Additional file 9: Figure S2. (TIF 721 kb)
Additional file 10: Which involves 2907 patients’ information across from the essential information such as age,gender,educational level, etc., to the variables including in our data analysis such as SUA levels, diagnosis of glycometabolism status, functional outcomes (poor or good) and so on. Each variable has its meaning and number 1,2,3 or more stands for the different meanings of each variable. For example, as for the variable “gender”, 1 stands for “male” and 2 stands for “female”. (XLS 912 kb)
Additional file 11: (PDF 29 kb)

## Abbreviations

ACROSS-China: The abnormal glucose regulation in patients with acute stroke across China study
BMI: Body mass index
CI: Confidential interval
D1 to D10: Decile1 to Decile 10
DM: Diabetes mellitus
EPCs: Endothelia progenitor cells
FPG: Fasting plasma glucose
HOMA2-IR: The Homeostasis Model Assessment 2-Insulin Resistance
ICH: Intracerebral hemorrhage
IFG: Impaired fasting glucose
IGT: Impaired glucose tolerance
mRS: Modified Rankin Scale
NIHSS: The National Institutes of Health Stroke Scale
NOACs: Non-VKA oral anticoagulants
OGTT: Oral glucose tolerance test
ORs: Odds ratios
Q1 to Q4: Quartile 1 to quartile 4
ROS: Reactive oxygen species
SAH: Subarachnoid hemorrhage
SUA: Serum uric acid
WHO: World Health Organization
XO: Xanthine Oxidase
