# Supplementary material for: Lower serum uric acid level strongly predict short-term poor functional outcome in acute stroke with normoglycaemia: a cohort study in China
Source: BMC Neurol. 2017 Feb 1;17:21. doi: 10.1186/s12883-017-0793-6 (PMC5286688; doi:10.1186/s12883-017-0793-6)
Supplement: Additional file 1: Table S3. — Aspirin use and SUA levels stratified by glycometabolism. (DOC 32 kb) [file 12883_2017_793_MOESM1_ESM.doc]

*Additional file 3: Table S3*. Aspirin use and SUA levels stratified by glycometabolism

| Serum uric acid level |  |  | Aspirin use(n=1542) |  |  |  |
| --- | --- | --- | --- | --- | --- | --- |
|  | DM(n=664) |  | PreDM(n=380) |  | Normal(n=498) |  |
|  | no | yes | no | yes | no | yes |
| <221umol/L | 99 | 35 | 38 | 20 | 55 | 35 |
| 221-288umol/L | 133 | 46 | 73 | 30 | 79 | 49 |
| 288-364umol/L | 124 | 55 | 78 | 21 | 99 | 34 |
| >364umol/L | 124 | 48 | 97 | 23 | 113 | 34 |
|
| P | 0.717 |  | 0.083 |  | 0.008 |  |
