# Supplementary material for: Lower serum uric acid level strongly predict short-term poor functional outcome in acute stroke with normoglycaemia: a cohort study in China
Source: BMC Neurol. 2017 Feb 1;17:21. doi: 10.1186/s12883-017-0793-6 (PMC5286688; doi:10.1186/s12883-017-0793-6)
Supplement: Additional file 2: Table S4. — Coumadin use and SUA levels stratified by glycometabolism. (DOC 31 kb) [file 12883_2017_793_MOESM2_ESM.doc]

*Additional file 4: Table S4.* Coumadin use and SUA levels stratified by glycometabolism

| Serum uric acid quartiles |  |  | Coumadin use(n=20) |  |  |  |
| --- | --- | --- | --- | --- | --- | --- |
|  | DM(n=7) |  | PreDM(n=6) |  | Normal(n=7) |  |
|  | no | yes | no | yes | no | yes |
| <221umol/L | 1 | 0 | 1 | 0 | 0 | 3 |
| 221-288umol/L | 1 | 0 | 2 | 0 | 1 | 0 |
| 288-364umol/L | 0 | 0 | 0 | 0 | 0 | 1 |
| >364umol/L | 5 | 0 | 2 | 1 | 1 | 1 |
|
| P | not available |  | 0.998 |  | 0.286 |  |
