# Supplementary material for: Lower serum uric acid level strongly predict short-term poor functional outcome in acute stroke with normoglycaemia: a cohort study in China
Source: BMC Neurol. 2017 Feb 1;17:21. doi: 10.1186/s12883-017-0793-6 (PMC5286688; doi:10.1186/s12883-017-0793-6)
Supplement: Additional file 3: Table S5. — Diuretics use and SUA levels stratified by glycometabolism. (DOC 31 kb) [file 12883_2017_793_MOESM3_ESM.doc]

*Additional file 5: Table S5*. Diuretics use and SUA levels stratified by glycometabolism

| Serum uric acid level |  |  | Diuretics use(n=124) |  |  |  |
| --- | --- | --- | --- | --- | --- | --- |
|  | DM(n=53) |  | PreDM(n=40) |  | Normal(n=31) |  |
|  | no | yes | no | yes | no | yes |
| <221umol/L | 11 | 5 | 5 | 1 | 8 | 2 |
| 221-288umol/L | 10 | 1 | 9 | 2 | 4 | 2 |
| 288-364umol/L | 15 | 1 | 8 | 0 | 3 | 2 |
| >364umol/L | 8 | 2 | 12 | 3 | 7 | 3 |
|
| P | 0.272 |  | 0.720 |  | 0.904 |  |
