# Supplementary material for: Lower serum uric acid level strongly predict short-term poor functional outcome in acute stroke with normoglycaemia: a cohort study in China
Source: BMC Neurol. 2017 Feb 1;17:21. doi: 10.1186/s12883-017-0793-6 (PMC5286688; doi:10.1186/s12883-017-0793-6)
Supplement: Additional file 4: Table S7. — SUA quartile and poor functional outcome at discharge with NIHSS adjusted as a continuous variable. (DOC 28 kb) [file 12883_2017_793_MOESM4_ESM.doc]

*Additional file 7: Table S7.* SUA quartile and poor functional outcome at discharge with NIHSS adjusted as a continuous variable

| Gender | SUA quartiles | Odds ratio with 95% confidence intervals | P |
| --- | --- | --- | --- |
| Total | <221umol/L | 4.54(1.34-12.41) | 0.015 |
|  | 221-288umol/L | 1.26(0.43-3.72) | 0.670 |
|  | 288-364umol/L | 1.23(0.46-3.29) | 0.685 |
|  | >364umol/L | ref |  |
| male | <221umol/L | 6.90(1.30-16.79) | 0.024 |
|  | 221-288umol/L | 0.82(0.22-3.06) | 0.772 |
|  | 288-364umol/L | 1.33(0.43-4.13) | 0.619 |
|  | >364umol/L | ref |  |
| female | <221umol/L | 12.90(0.87-39.56) | 0.153 |
|  | 221-288umol/L | 7.52(0.21-20.45) | 0.240 |
|  | 288-364umol/L | 0.69(0.12-19.65) | 0.844 |
|  | >364umol/L | ref |  |
