# Supplementary material for: Lower serum uric acid level strongly predict short-term poor functional outcome in acute stroke with normoglycaemia: a cohort study in China
Source: BMC Neurol. 2017 Feb 1;17:21. doi: 10.1186/s12883-017-0793-6 (PMC5286688; doi:10.1186/s12883-017-0793-6)
Supplement: Additional file 5: Table S6. — Multivariate analysis on SUA and poor functional outcome in normoglycaemic stroke stratified by gender. (DOC 22 kb) [file 12883_2017_793_MOESM5_ESM.doc]

*Additional file 6: Table S6*. Multivariate analysis on SUA and poor functional outcome in normoglycaemic stroke stratified by gender

| Gender | SUA quartiles | Odds ratio with 95% confidence intervals | P |
| --- | --- | --- | --- |
| male | <221umol/L | 5.06(1.17-12.05) | 0.031 |
|  | 221-288umol/L | 0.89(0.28-2.92) | 0.857 |
|  | 288-364umol/L | 1.03(0.38-2.90) | 0.954 |
|  | >364umol/L | ref |  |
| female | <221umol/L | 9.21(0.98-38.66) | 0.158 |
|  | 221-288umol/L | 6.85(0.68-18.56) | 0.243 |
|  | 288-364umol/L | 1.57(0.35-9.65) | 0.098 |
|  | >364umol/L | ref |  |
