# Supplementary material for: Lower serum uric acid level strongly predict short-term poor functional outcome in acute stroke with normoglycaemia: a cohort study in China
Source: BMC Neurol. 2017 Feb 1;17:21. doi: 10.1186/s12883-017-0793-6 (PMC5286688; doi:10.1186/s12883-017-0793-6)
Supplement: Additional file 6: Table S1. — Uric acid level and neurological improvement stratified by glycometabolism status. (DOC 31 kb) [file 12883_2017_793_MOESM6_ESM.doc]

*Additional file 1: Table S1.* Uric acid level and neurological improvement stratified by glycometabolism status

| Serum uric acid level |  |  | neurological improvement |  |  |  |
| --- | --- | --- | --- | --- | --- | --- |
|  | DM(n=1220) |  | Prediabetes(n=777) |  | Normal(n=910) |  |
|  | no | yes | no | yes | no | yes |
| <221umol/L | 176 | 119 | 82 | 99 | 108 | 114 |
| 221-288umol/L | 217 | 86 | 130 | 67 | 119 | 98 |
| 288-364umol/L | 201 | 105 | 116 | 72 | 122 | 88 |
| >364umol/L | 178 | 108 | 109 | 86 | 129 | 117 |
|
| P | 0.015 |  | <0.001 |  | 0.246 |  |
