# Supplementary figures and images for: Lower serum uric acid level strongly predict short-term poor functional outcome in acute stroke with normoglycaemia: a cohort study in China
Source: BMC Neurol. 2017 Feb 1;17:21. doi: 10.1186/s12883-017-0793-6 (PMC5286688; doi:10.1186/s12883-017-0793-6)

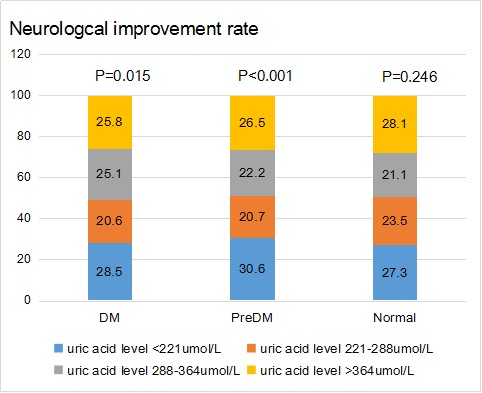

Supplement: Additional file 7: Figure S1. — (TIF 740 kb) [file 12883_2017_793_MOESM7_ESM.tif]

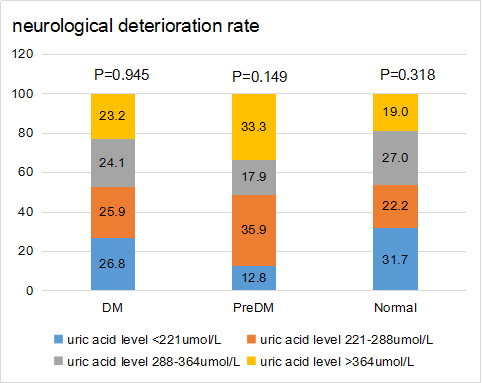

Supplement: Additional file 9: Figure S2. — (TIF 721 kb) [file 12883_2017_793_MOESM9_ESM.tif]
