# Supplementary material for: Lower serum uric acid level strongly predict short-term poor functional outcome in acute stroke with normoglycaemia: a cohort study in China
Source: BMC Neurol. 2017 Feb 1;17:21. doi: 10.1186/s12883-017-0793-6 (PMC5286688; doi:10.1186/s12883-017-0793-6)
Supplement: Additional file 8: Table S2. — Uric acid level and neurological deterioration stratified by glycometabolism status. (DOC 31 kb) [file 12883_2017_793_MOESM8_ESM.doc]

***Additional file 2: Table S2***. Uric acid level and neurological deterioration stratified by glycometabolism status

| Serum uric acid level |  |  | Neurological deterioration |  |  |  |
| --- | --- | --- | --- | --- | --- | --- |
|  | DM(n=1220) |  | Prediabetes(n=777) |  | Normal(n=910) |  |
|  | no | yes | no | yes | no | yes |
| <221umol/L | 264 | 30 | 175 | 5 | 202 | 20 |
| 221-288umol/L | 273 | 29 | 183 | 14 | 203 | 14 |
| 288-364umol/L | 279 | 27 | 181 | 7 | 192 | 17 |
| >364umol/L | 259 | 26 | 182 | 13 | 233 | 12 |
|
| P | 0.945 |  | 0.149 |  | 0.318 |  |
