# Supplementary material for: Lower serum uric acid level strongly predict short-term poor functional outcome in acute stroke with normoglycaemia: a cohort study in China
Source: BMC Neurol. 2017 Feb 1;17:21. doi: 10.1186/s12883-017-0793-6 (PMC5286688; doi:10.1186/s12883-017-0793-6)
Supplement: Additional file 11: — (PDF 29 kb) [file 12883_2017_793_MOESM11_ESM.pdf]

SUPPLEMENTAL MATERIAL

Abnormal glucose regulation in inpatients with acute stroke across China－ a national multicenter prospective study (ACROSS-CHINA)

Principal investigator: Yongjun Wang.

Steering committee: Yongjun Wang, Qiang Dong, Liying Cui, Shengnian Zhou, Fengchun Yu, Xingquan Zhao, Chunxue Wang.

Study organization executive committee: Huaguang Zheng, Qian Jia, Yilong Wang, Yong zhou, Liping Liu, Jing Jing.

Statistician: Gaifen Liu.

Data monitoring committee: Zhijiang Wang, Haibo Wu.

Clinical Centers (site principal investigator):

Yongjun Wang, Beijing Tiantan Hospital; Yuheng Sun, Beijing Jishuitan Hospital; Fengchun Yu, Beijing Haidian Hospital; Huashan Sun, General Hospital of CNPC in jilin; Jinying Li, Jilin Oilfield General Hospital; Guozhong Li, First Affiliated Hospital of Harbin Medical University; Li Guo, Hebei Medical University Second Hospital; Yibin Cao, Tangshan Gongren Hospital; Xiaodong Yuan, Tangshan Kailuan hospital; Juntao Li, Handan Central Hospital; Yiping Wu, First hospital of Handan City; Jiewen Zhang, Henan Province People's Hospital; Yuming Xu, First Affiliated Hospital of Zhengzhou University; Shengnian Zhou, Qilu Hospital of Shandong Province; Guanglai Li, Second Affiliated Hospital of Shanxi University Medical College; Minxia Guo, Shanxi Provincial Peoples Hospital; Shuguang Li, People's

Hospital of Bozhou; Shenggang Sun, Wuhan Union Hospital; Wei Wang, Tongji Hospital of Tongji Medical College of HUST; Bo Xiao, Xiangya Hospital; Renbin Huang, First People's Hospital of Chenzhou City; Kangning Chen, Southwest Hospital; Xiaoping Wu, Sichuan Mianyang City people's Hospital; Qingke Bai, Shanghai Pudong New Area People's Hospital; Xiaojiang Sun, Sixth Affiliated Hospital of Shanghai Jiaotong University School of Medicine; Xin Li, Shanghai Yangpu District Central Hospital; Dexiang Gu, Shanghai East Hospital; Benyan Luo, First Affiliated Hospital of Medical College of Zhejiang University; Rongyuan Zheng, First Affiliated Hospital of Wenzhou Medical College; Yefeng Cai, Traditional Chinese Medicine Hospital of Guangdong Province; Xuean Mo, First Affiliated Hospital of Guangxi Medical University; Li Gao, Third People's Hospital of Chengdu; Xiaoping Gao, Hunan Province People's Hospital; Chunbo Qi, The people's Hospital of Penglai city Shandong Province; Lianbo Gao, Fourth Affiliated Hospital of China Medical University; Jianping Niu, Second people's Hospital of Xiamen City; Yi Wu, Yiwu Central Hospital; Yimin Shan, First Hospital of Nanchang City.
